# Hydroxytyrosol Prevents Doxorubicin-Induced Oxidative Stress and Apoptosis in Cardiomyocytes

**DOI:** 10.3390/antiox11061087

**Published:** 2022-05-30

**Authors:** Ivana Sirangelo, Maria Liccardo, Clara Iannuzzi

**Affiliations:** Department of Precision Medicine, Università degli Studi della Campania “Luigi Vanvitelli”, Via L. De Crecchio 7, 80138 Naples, Italy; ivana.sirangelo@unicampania.it (I.S.); maria.liccardo@unicampania.it (M.L.)

**Keywords:** Doxorubicin, ROS, hydroxytyrosol, chemopreventive agents, antioxidant, cardiotoxicity

## Abstract

Doxorubicin (Dox) is a highly effective chemotherapeutic agent employed in the handling of hematological and solid tumors. The effective use of Dox in cancer therapy has been seriously limited due to its well-known cardiotoxic side effects, mainly mediated by oxidative damage. Therefore, the identification of an effective and safe antagonist against Dox-induced cardiotoxicity remains a challenge. In this respect, as plant polyphenols have attracted considerable interest due to their antioxidant properties and good safety profile, hydroxytyrosol (HT), the major phenolic compound in olive oil, could be a potential candidate due to its remarkable antioxidant and anticancer powers. In this study, the effect of HT was tested on Dox-induced cardiotoxicity by using a combination of biochemical and cellular biology techniques. Interestingly, HT was able to counteract Dox-induced cytotoxicity in cardiomyocytes by acting on the SOD2 level and the oxidative response, as well as on apoptotic mechanisms mediated by Bcl-2/Bax. At the same time, HT did not to interfere with the antitumorigenic properties of Dox in osteosarcoma cells. This study identifies new, beneficial properties for HT and suggests that it might be a promising molecule for the development of additional therapeutic approaches aimed at preventing anthracycline-related cardiotoxicity and improving long-term outcomes in antineoplastic treatments.

## 1. Introduction

Doxorubicin (Dox) is an effective anthracycline chemotherapeutic agent widely used in the treatment of several types of cancer, including solid and haematological malignancies. Indeed, by interfering with both DNA synthesis and replication, Dox promotes cancer cell death in several ways, mainly via topoisomerase II poisoning and DNA base pair intercalation [1,2]. Nevertheless, the success rate in first-line is generally followed by a strong reduction in responsiveness and other collateral effects, which hinder further dose intensification and restrict its use in clinical practice [3,4,5,6]. In particular, Dox is known to be responsible for cumulative and dose-dependent cardiotoxicity, which results in increased risks of mortality among cancer patients, thus limiting its wide clinical applications [7]. Several mechanisms have been proposed for Dox-induced cardiotoxicity and heart failure: oxidative stress, free radical generation and apoptosis are the main mechanisms involved. Moreover, DNA damage, calcium homeostasis alteration, inflammatory response, mitochondrial dysfunction and autophagy have also been reported to play a pivotal role [6,8,9,10,11]. Therefore, the generation of reactive oxygen species (ROS) upon Dox treatment is believed to be the key event underlying the interconnected intracellular signaling pathways leading to cardiotoxicity. Cardiac cells are very sensitive to oxidative stress considering their low antioxidant defenses; antioxidant enzymes, especially catalases, are often deficient in counteracting ROS production during Dox administration [12,13].

**Figure 1:**
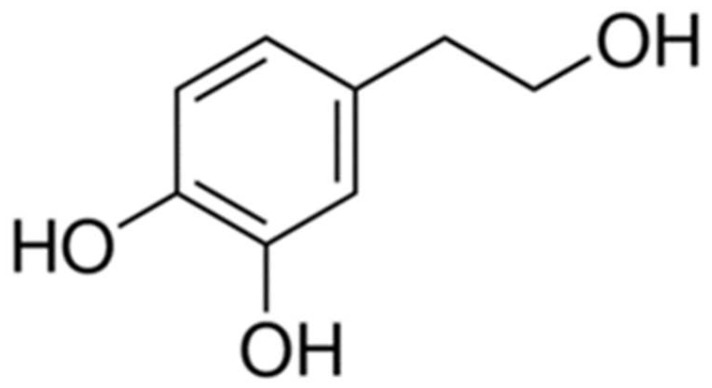
Chemical structure of hydroxytyrosol (HT).

In order to prevent Dox-induced cardiotoxicity, several approaches have been suggested, including the use of new, less toxic Dox analogs, the application of high-sensitivity diagnostic procedures and cardioprotective supplemental therapies. Currently, early preventive treatment with additional cardioprotective agents is strongly recommended to counteract cardiotoxicity in patients undergoing oncological treatment [14,15,16,17]. Several therapeutic strategies to prevent Dox-induced cardiotoxicity have also been explored in preclinical studies. Notably, angiotensin receptor blockers and beta blockers have shown beneficial effects, though these effects remain controversial [18,19,20,21]. Dexrazoxane is the only FDA-approved agent for preventing Dox-induced cardiac toxicity, although it has been reported to induce secondary malignancies and a concomitant reduction in anticancer efficacy [22,23,24,25]. Thus, alternative treatment strategies with lower or no toxic side effects are currently being investigated. In this respect, plant-derived polyphenols have been found to counteract Dox-induced cardiotoxicity due to their antioxidant, anti-inflammatory and anti-apoptotic properties [26,27,28,29,30,31,32]. Recently, particular interest has been paid to polyphenols, highly concentrated in olive oil, including oleuropein aglycone and its main metabolite, hydroxytyrosol (3,4-dihydroxyphenylethanol, HT), for their antioxidant and anti-inflammatory power [33,34,35,36,37,38] (Figure 1). Specifically, it has been reported that HT is able to ameliorate Dox-associated chronic cardiac toxicity in rats with breast cancer by reducing oxidative stress and mitochondrial disfunction [39]. In this study, using a cardiomyocyte in vitro model, we have identified a beneficial role for HT in Dox-induced cardiotoxicity and analyzed the molecular mechanisms involved in HT protection. Our data show that HT is able to counteract Dox-induced cytotoxicity in cardiomyocytes by acting on ROS levels and the oxidative response, as well as on apoptotic mechanisms mediated by Bcl-2/Bax. In addition, we have tested the effect of HT in Dox-treated osteosarcoma cells to check for a possible concomitant reduction in anticancer efficacy.

## 2. Materials and Methods

### 2.1. Materials

Doxorubicin (#5927, cell signaling), hydroxytyrosol (H4291 Sigma-Aldrich, Missouri, MO, USA), 3-(4,5-dimethylthiazol-2-yl)-2,5-diphenyl-tetrazoliumbromide (MTT) (Sigma-Aldrich). Antibodies: anti-cleaved caspase-3 (#9662), anti-SOD2 (#13194), anti-Bax (#2774), anti-Bcl-2 (#15071), anti-phospho-p38 MAPK (#9211), anti-p-38 (#9212), anti-phospho-histone H2AX (Ser139) (#80312), Anti-GAPDH (#97166), anti-tubulin (#2146) (Cell Signalling Technology, Danvers, MA, USA). Secondary antibodies: anti-mouse IgG (#7076), anti-rabbit IgG (#7074) (Cell Signalling Technology).

### 2.2. Cell Cultures and Treatments

Embryonic rat-cardiac-tissue-derived H9c2 cardiomyoblasts (ATCC^®^ CRL-1446) and human U2OS osteosarcoma cells (ATCC^®^ CRL-1446) were purchased from ATCC (Manassas, VA, USA) and cultured in Dulbecco’s Minimum Essential Medium (DMEM) (AL007, Microgem, Naples, Italy) supplemented with 10% FBS, 2.0 mM glutamine (X0550, Microgem), 100 units/mL penicillin and 100 mg/mL streptomycin (A001, Himedia, Maharashtra, India) in a 5.0% CO_2_ humidified environment at 37 °C. Cells were grown for 18 h before treatments. The experimental groups were: CTR: untreated cells; Dox: cells treated with Doxorubicin 0.1 μM; Dox–HT: cells cotreated with 50 μM HT and Doxorubicin 0.1 μM; HT: cells treated with HT 50 μM.

### 2.3. MTT Assay

Cell viability was assessed through the ability of cells to reduce the metabolic dye 3-[4,5-dimethylthiazol-2-yl]-2,5-diphenyltetrazolium bromide (MTT) to a blue formazan product [40,41]. After treatments, cells were incubated with 0.5 mg/mL MTT in cell medium and incubated for 3 h at 37 °C. After removing the medium, cells were treated with isopropyl alcohol, 0.1 M HCl for 20 min. Levels of reduced MTT were assayed by measuring the difference in absorbance between 570 and 690 nm. Data are expressed as percentage reduction of MTT with respect to the control ± S.D. Data were obtained from five independent experiments carried out in triplicate.

### 2.4. Detection of Intracellular ROS

Intracellular ROS were detected by means of an oxidation-sensitive fluorescent probe 2′,7′-dichlorofluorescin diacetate (DCFH-DA) [42,43]. Cells were grown in 12-well plates, preincubated with DCFH-DA for 30 min before treatment. Control experiments were performed using untreated cells and cells exposed to 1 mM H_2_O_2_. After incubation, cells were washed twice with PBS buffer and then lysed with Tris-HCl 0.5 M, pH 7.6, 1% SDS. The nonfluorescent DCFH-DA was converted by oxidation to the fluorescent molecule 2′,7′-dichlorofluorescein (DCF). DCF fluorescence intensity was quantified on a Perkin Elmer Life Sciences LS 55 spectrofluorometer using an excitation wavelength of 488 nm and an emission wavelength of 530 nm. Data are expressed as average ± S.D. from five independent experiments carried out in triplicate.

### 2.5. Immunoblotting

Cells from different experimental groups were collected by centrifugation, resuspended in lysis buffer (10 mM Tris pH 8.0, 150 mM NaCl, 10 mM NaF, 1 mM dithiothreitol, 1% NP-40), along with the protease inhibitor cocktail and allowed to swell on ice for 20 min. The supernatant was taken after centrifugation at 13,000× *g* at 4 °C for 30 min, and protein concentration was estimated using Bradford’s reagent (BioRad, Hercules, CA, USA). Protein extracts (25 µg) were separated by 12% SDS-PAGE under reducing conditions and blotted onto a polyvinylidene difluoride membrane in transfer buffer (25 mM Tris, 192 mM glycine, 20% methanol, 0.1% SDS). The blots were then probed with indicated primary antibodies, followed by the corresponding horseradish peroxidase (HRP)-conjugated secondary antibodies. Immunoreactive bands were visualized using an enhanced chemiluminescence detection kit (Elabscience, Houston, TX, USA) with Chemi Doc XR (Biorad, Hercules, CA, USA). Densitometric analysis was performed using ImageJ software, processing the digital image and converting the intensity of every protein band in an arithmetic value. Data analysis was performed by comparing each sample with the control, and normalization was achieved using the housekeeping gene.

### 2.6. Trypan Blue Assay

Trypan Blue assay was performed to estimate the number of dead cells in the experimental groups. Trypan Blue is a cell membrane-impermeable dye and, therefore, its presence inside cells is indicative of damaged membranes. After a 48-h treatment, cells were collected and cell counting was performed by mixing 10 μL of cell suspension with an identical volume of Trypan Blue (0.4%, *v/v*). Trypan Blue, when inside cells, renders the cells dark blue, making it possible to selectively quantify dead cells. For U2OS experiments, besides the number of blue-stained cells (dead cells), not-stained cells (viable cells) were also quantified. Trypan Blue experiments were performed three times in replicates of six wells for each datapoint in each experiment. Data are presented as means ± standard deviation for a representative experiment.

### 2.7. Statistical Analysis

Statistical analyses were performed using Stata software (Version 13.0; StataCorp LP., College Station, TX, USA). Tukey’s post hoc test was used if the treatment was significant upon analysis of variance (ANOVA). All data are represented as the mean ± SE. Statistical significance was set at *p* < 0.05.

## 3. Results

### 3.1. HT Markedly Reduces Dox-Induced Toxicity in H9c2 Cells

**Figure 2:**
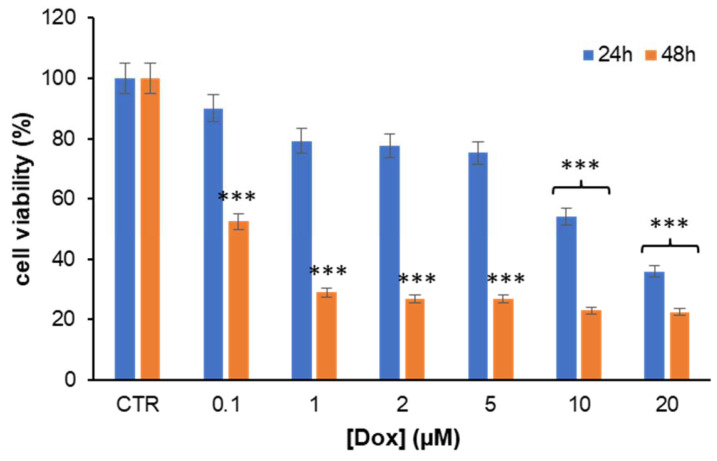
Evaluation of Dox cytotoxicity in cardiomyoblasts. Cell viability was evaluated by MTT assay in H9c2 cells exposed for 24 and 48 h to an increasing concentration of Dox (from 0.1 to 20 μM). CTR: untreated cells. Data are expressed as average percentage of MTT reduction ± SD relative to untreated cells from triplicate wells from 5 separate experiments. Other experimental details are described in the Materials and Methods section. *** *p* ˂ 0.001 versus CTR.

Before addressing the potential impact of combination HT plus Dox treatment in a cardiac cell model, first, the toxicity of Dox was tested in H9c2 cells, a widely accepted representative cardiac cell line, in order to optimize the experimental conditions. Specifically, H9c2 cells were exposed to different concentrations of Dox (0.1–20 μM), and the cell viability was assessed in 24- and 48-h treatments by MTT assay (Figure 2). As expected, cells exposed to Dox showed a clear reduction of the cell viability at any concentration tested. In particular, in the 24-h treatment, significative cytotoxicity was observed only in the presence of 10 and 20 µM Dox, whereas, in the 48-h treatment, a marked decrease in cell viability was observed at any Dox concentration. Indeed, after 48 h of incubation, very high toxicity (about 70% reduction) was recorded in the range 1–20 µM Dox, while lower toxicity (about 50% reduction) was observed in the presence of 0.1 µM Dox (Figure 2).

**Figure 3:**
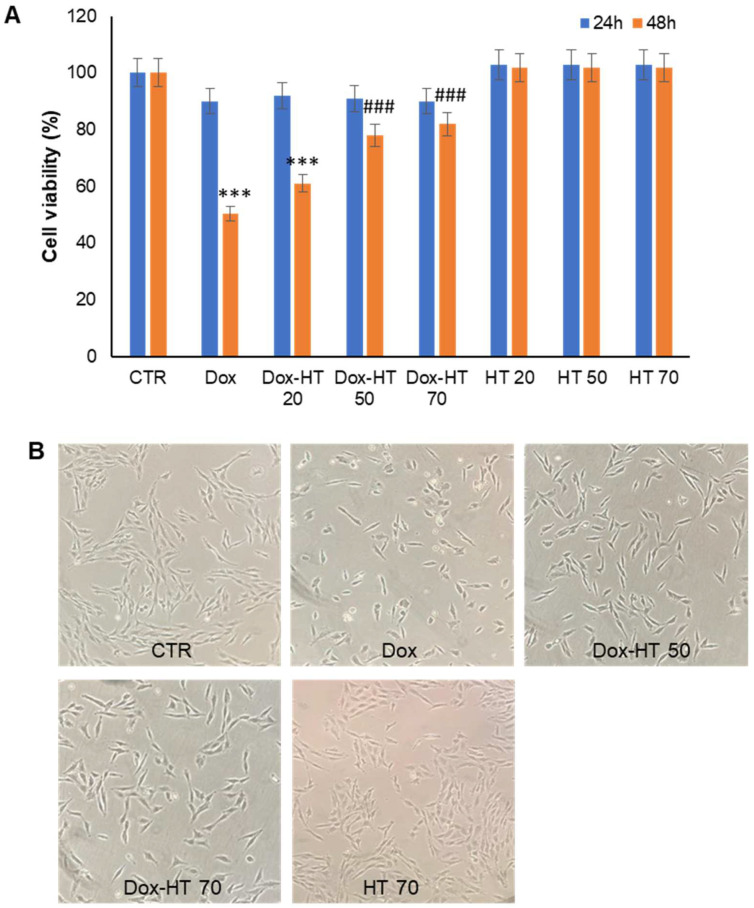
Effect of HT on Dox-induced cytotoxicity. (**A**) Cell viability was evaluated by MTT assay in H9c2 cells exposed to 0.1 µM Dox (Dox), and cotreated with 20 (Dox-HT 20), 50 (Dox-HT 50) and 70 (Dox-HT 70) µM HT for 24 and 48 h. Data are expressed as average percentage of MTT reduction ± SD relative to untreated cells (CTR) from triplicate wells from 5 separate experiments. *** *p* ˂ 0.001 versus CTR, ### *p* ˂ 0.001 versus Dox. (**B**) Phase contrast microscopy images (magnification 10×) of H9c2 cells after 48-h incubation. CTR: untreated cells; Dox: cells exposed to 0.1 µM Dox; Dox–HT 50/70: cells cotreated with 50 and 70 µM of HT. Other experimental details are described in the Materials and Methods section.

On the basis of the above results, the working concentration for our study was set at 0.1 µM Dox in order to minimize the cell death in the 48-h treatment. To investigate the effect of HT in Dox-induced cytotoxicity, H9c2 cells were cotreated with Dox and HT at different concentrations, and the cell viability was monitored by MTT assay the 24- and 48-h treatments (Figure 3A).

Interestingly, while cells treated with Dox showed a marked reduction (about 50%) in cell viability in the 48-h treatment, no toxicity was observed for cells treated in the presence of HT in the range 50–70 µM (about 80% cell viability). Indeed, while the coincubation with 20 µM HT only slightly affected the Dox cytotoxicity, the presence of 50 and 70 µM HT was able to protect cells from Dox cardiotoxicity. As in some cases, HT showed higher efficiency when preincubated some hours before treatment; in this experiment, HT was also preincubated with cells 12 h before Dox treatment, but no difference was observed compared to the co-incubated Dox–HT cells. In addition, to test the efficacy of the HT protective effect even at higher Dox concentrations, cell viability was monitored using 10 µM Dox as a working concentration, and a strong protective effect was observed for 50 and 70 µM HT in both the 24- and 48-h cotreatments (data not shown). The different experimental groups were also analyzed by phase-contrast microscopy in order to evaluate modifications in cell morphology and cell number upon treatment after 48 h (Figure 3B). Figure 3B shows that cells incubated with Dox for 48 h were showed both modifications in cell morphology and reduction in the cell number, whereas those co-incubated with both 50 and 70 µM HT showed reduced qualitative and quantitative alterations. These data clearly suggest that 50 µM HT reduces Dox-induced toxicity in H9c2 cardiomyoblasts.

### 3.2. HT Prevents Dox-Induced ROS Production and SOD2 Activation in H9c2 Cells

**Figure 4:**
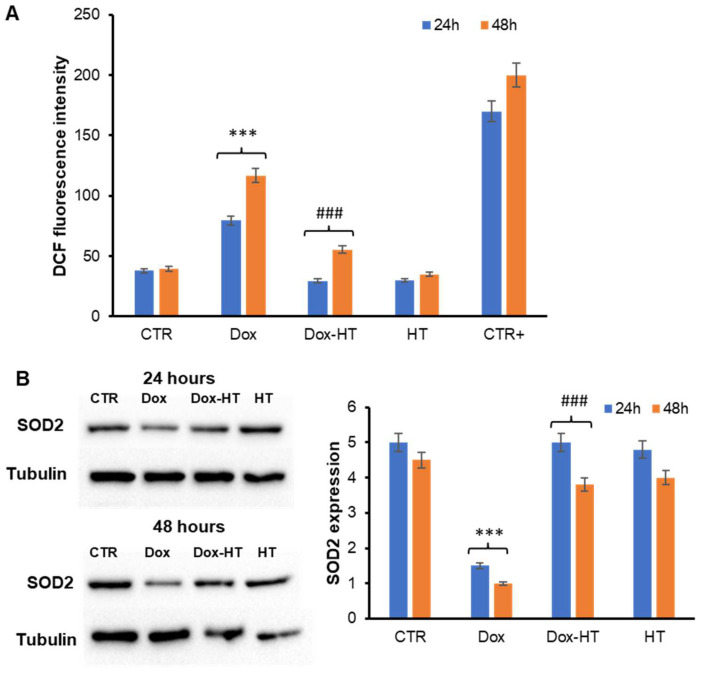
Role of HT in Dox-induced oxidative stress. H9c2 cells were exposed to 0.1 µM Dox (Dox) and co-incubated with 50 µM HT (Dox–HT), and both ROS production by DCFH-DA assay (**A**) and SOD2 expression by western-blot analysis (**B**) were evaluated. CTR: untreated cells; HT: cells treated with HT only; CTR+: cells treated with 1.0 mM H_2_O_2_. Data are expressed as average ± S.D. from five independent experiments carried out in triplicate. Other experimental details are described in the Materials and Methods section. *** *p* ˂ 0.001 versus CTR, ### *p* ˂ 0.001 versus Dox.

Oxidative stress is one of the primary mechanisms of Dox-induced cardiotoxicity, prompted by the excessive generation of ROS and subsequent mitochondrial membrane depolarization [44,45]. Conversely, HT is known to exert its beneficial properties through strong antioxidant action [36,46]. In this respect, to identify the molecular basis of the cellular protection by which HT counteracts Dox cardiotoxicity, evaluated the effect of HT on Dox-associated oxidative damage was evaluated. First, the ability of HT to reduce ROS production associated with Dox treatment was tested (Figure 4).

In particular, as Dox is documented to induce ROS production in H9c2 cardiomyoblasts, the intracellular ROS levels in H9c2 cells co-incubated with HT were tested using a DCFH-DA fluorescence assay (Figure 4A). Interestingly, while Dox promotes ROS production both at 24- and 48-h incubation periods, as indicated by the increase in the DCF fluorescence, in the sample co-incubated with HT, the ROS levels were similar to untreated cells, thus suggesting that HT is able to counteract Dox-induced oxidative stress in H9c2 cells. Evaluation of oxidative stress was also performed through the estimation of SOD2 expression, a key mediator of ROS production, by Western blot analysis in H9c2 cells incubated for 24 and 48 h (Figure 4B). As expected, cells treated with Dox showed a reduced expression of SOD2 associated with an increase in oxidative stress in both the 24- and 48-h incubations. Conversely, SOD2 levels in cells cotreated with both Dox and HT were similar to that of untreated cells, further confirming that the presence of HT is able to counteract the Dox-dependent oxidative stress in H9c2 cells.

### 3.3. HT Protects H9c2 Cells by the Dox-Induced Apoptosis

**Figure 5:**
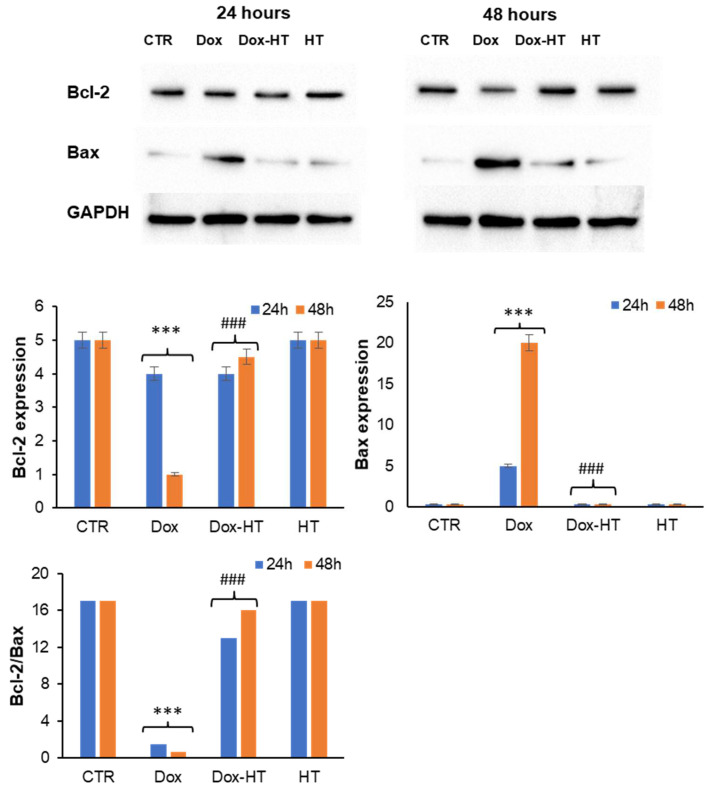
Role of HT in Dox-induced apoptosis. Western blot analysis of Bcl2 and Bax in H9c2 cells exposed to 0.1 μM Dox (Dox) and co-treated with 50 μM HT (Dox-HT). CTR: untreated cells. The images are representative of Western blotting from three different cellular extracts with similar results. Other experimental details are described in the Materials and Methods section. *** *p* ˂ 0.001 versus CTR, ### *p* ˂ 0.001 versus Dox.

Dox-induced cardiotoxicity is characterized by an increase in oxidative stress that triggers the activation of apoptosis in cardiomyocytes [47,48]. This process is mediated by the release of cytochrome C (Cyt c) from the mitochondria into the cytosol, where it triggers the caspase cascade of apoptosis activation, which ultimately leads to the death of cardiomyocytes and the impairment of the contractile function of the heart [5,49]. The release of Cyt c is regulated by members of the B-cell lymphoma 2 (Bcl-2) family, which consists of both pro-apoptotic Bcl-2-like protein 4 (Bax) and anti-apoptotic (Bcl-2) proteins [50]. Bax is located in the cytosol and, when activated, it translocates to the outer mitochondrial membrane, depolarizing it and stimulating the release of Cyt c into the cytosol, whereas Bcl-2 inhibits the release of Cyt c from the mitochondria [51,52]. Once released, Cyt c in the cytosol is responsible for the activation of caspase 3 (through p38) and subsequent cellular apoptosis [53]. It has been reported that Dox can induce cell death by apoptosis in cardiomyocytes through a decrease in the Bcl-2/Bax ratio responsible for caspase 3 activation [27,48,54]. Thus, to further investigate HT-mediated cell death reduction in response to Dox treatment, H9c2 cells were analyzed using Western blotting analysis to identify putative effects of HT in Dox-induced apoptosis. First, the expression of Bcl-2 and Bax in H9c2 cells in the presence and absence of HT upon Dox-treatment after 24 and 48 h was evaluated (Figure 5). As expected, the incubation with Dox promoted a decrease of anti-apoptotic Bcl-2 protein expression and an increase in the pro-apoptotic Bax after a 48-h incubation. Conversely, in cells co-incubated with HT, no variation in the Bcl-2/Bax ratio was observed, thus suggesting that the presence of HT is able to interfere with the Bax pro-apoptotic pathway (Figure 5).

**Figure 6:**
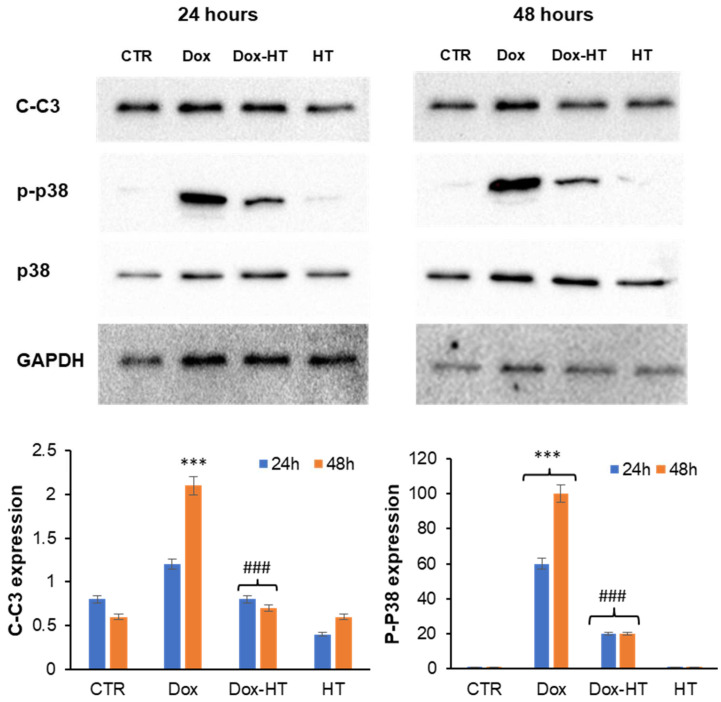
Role of HT in Dox-induced apoptosis. Western blot analysis of cleaved caspase-3 (C-C3) and phosphorylated p38 (p-p38) in H9c2 cells exposed to 0.1 μM Dox (Dox) and co-treated with 50 μM HT (Dox-HT). CTR: untreated cells. The p38 phosphorylation was quantified through the evaluation of the p-p38/p38 ratio. The images are representative of Western blotting from three different cellular extract with similar results. Other experimental details are described in the Materials and Methods section. *** *p* ˂ 0.001 versus CTR, ### *p* ˂ 0.001 versus Dox.

To further confirm this hypothesis, the activation of caspase 3 in the same experimental groups was also evaluated by measuring the cleaved active caspase 3 (C-C3) and the phosphorylated p38 MAP kinase, directly linked to caspase 3 activation (Figure 6). In this case, while Dox promoted both caspase 3 cleavage and p38 phosphorylation after 48 h, no activation was observed in cells co-incubated with HT, thus suggesting that HT is able to counteract Dox-induced apoptosis in H9c2 cells.

### 3.4. HT Effect in DNA Damage Dox-Induced in H9c2 Cells

**Figure 7:**
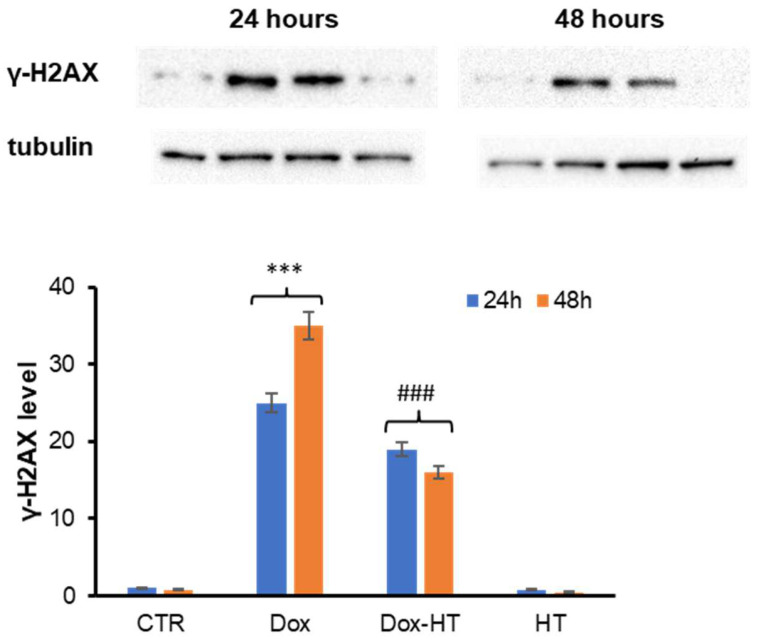
Role of HT in Dox-induced DNA damage. Western blot analysis of phospho-histone H2AX (γ-H2AX) in H9c2 cells exposed to 0.1 μM Dox (Dox) and co-treated with 50 μM HT (Dox-HT). CTR: untreated cells. The images are representative of Western blotting from three different cellular extracts with similar results. Other experimental details are described in the Materials and Methods section. *** *p* ˂ 0.001 versus CTR, ### *p* ˂ 0.001 versus Dox.

Dox-induced cardiotoxicity is believed to be associated with the redox cycling activity responsible for ROS production, as well as to Top2-targeting activity, which produces Top2–DNA covalent complexes; both related activities are responsible for DNA damage [55,56]. In this respect, we tested whether HT could prevent Dox-induced DNA damage in H9c2 cardiomyocytes through its antioxidant activity. To this end, the level of phospho-histone H2AX (γ-H2AX), a key marker of DNA damage, was evaluated through Western blot analysis in cells treated with Dox in the presence and absence of HT for 24 and 48 h (Figure 7). As expected, Dox promoted a clear increase in the γ-H2AX band in both the 24- and 48-h treatments. Interestingly, in samples co-incubated with Dox and HT, the level of γ-H2AX was reduced, suggesting that HT is able to counteract Dox-induced DNA damage in H9c2 cells.

### 3.5. HT Does Not Impair Antitumoral Dox Activity in Osteosarcoma Cells

**Figure 8:**
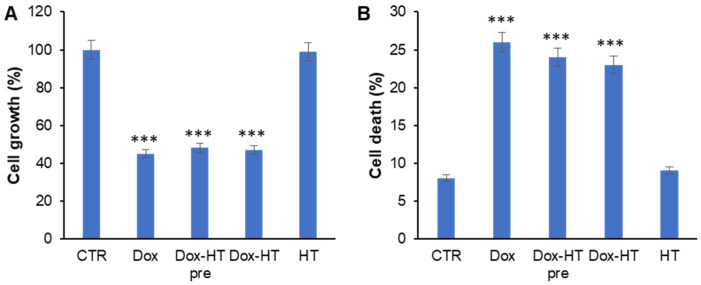
Effect of HT on Dox-induced antineoplastic properties in U2OS osteosarcoma cells. Number of living (**A**) and dead (**B**) cells for U2OS cells exposed to 0.1 μM Dox for 48 h, pretreated (Dox-HT pre) and co-treated (Dox-HT) with 50 μM HT. CTR: untreated cells; Dox: cells exposed to 0.1 μM Dox; Dox–HT pre: HT pretreated cells for 18 h; Dox–HT: HT co-treated cells. HT: cells exposed to 50 μM HT. Other experimental details are described in the Materials and Methods section. *** *p* ˂ 0.001 versus CTR.

Despite the high number of side effects observed in treatments, including those that are potentially lethal, such as the cardiotoxicity, Dox is still considered an elective therapy for several tumor types, such as breast cancer and osteosarcoma [57,58]. To highlight the protective effect of HT in Dox-induced cardiotoxicity and, at the same time, to exclude the possible impairment by HT of Dox antitumor activity, the effects of HT on the antitumoral action of Dox were also evaluated. To this end, the effect of Dox and HT in different combinations were tested in U2OS osteosarcoma cells, a widely used cell model that is very sensitive to Dox [59]. In particular, U2OS cells were treated with 0.1 µM Dox in the presence and absence of 50 µM HT for 48 h, and cell viability was evaluated through the estimation of living and dead cells (Figure 8). As expected, in cells treated with only Dox, strong decrease in living cells (−55%) and a 3-fold increase in dead cells was observed, whereas HT did not affect both cell growth and cell death compared to the control. Interestingly, both HT combinatory treatments caused live cell reduction and cell death increase comparable to Dox alone, thus suggesting that HT does not interfere with the antineoplastic properties of Dox in osteosarcoma cells. Moreover, analysis of ROS production in the same experimental groups revealed that Dox does not induce oxidative stress in osteosarcoma cells, both in the presence and absence of HT (data not shown). This might explain the inability of HT to interfere with the anticancer activity of Dox.

## 4. Discussion

Despite potentially lethal late cardiotoxicity, Dox is still considered a preferential treatment for several tumors in which no target therapy has been identified [57,58]. Therefore, mitigating Dox-related cardiac issues is considered a long-standing target in clinical oncology, and it is strongly needed in order to extend the duration of action and, consequently, Dox antitumoral efficacy. For this purpose, several different Dox analogs and formulations have been developed, and combination therapy has been suggested to be the best approach for treating cancer patients in an advanced state with lower or no toxic side effects.

Dox-induced cardiomyopathy is due to several mechanisms, among them, ROS overproduction and apoptosis remain the hallmark of Dox cardiotoxicity [5,6,27,46,60]. In this respect, several plant-derived polyphenols have drawn attention in pharmaceutical applications due to their ability to reduce ROS production and accumulation in the human body [26,27,28,29,30,31,32,61,62,63]. Recently, it has been reported that HT, the major phenolic compound in olive oil, is able to counteract Dox-associated chronic cardiac toxicity in rats with breast cancer by reducing oxidative stress and mitochondrial disfunction [39].

In the present study, we show that HT significantly counteracts Dox-induced toxicity in H9c2 cardiomyoblasts. In particular, HT is able to restore cell viability and damage following Dox exposure, as indicated by reductions in cell viability and morphological analysis.

**Figure 9:**
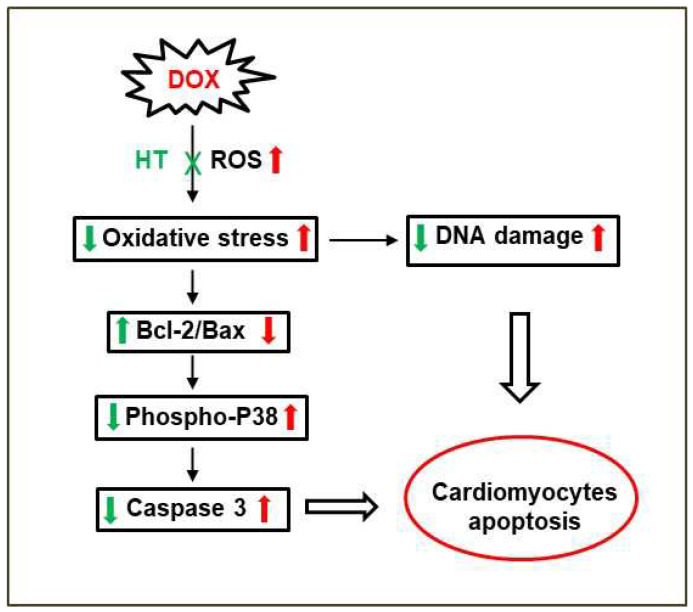
Working hypothesis on the role of HT in Dox-induced cardiotoxicity.

In order to identify the molecular basis of the cellular protection by which HT counteracts Dox cardiotoxicity, first, we have evaluated the effect of HT on Dox-associated oxidative damage. Interestingly, while Dox promotes ROS production and SOD2 expression in cardiomyocytes, the co-incubation with HT restores both ROS and SOD2 levels, thus suggesting that HT is able to counteract Dox-induced oxidative stress. In Dox-induced cardiotoxicity, the increase in oxidative stress promotes the release of cytochrome C from the mitochondria into the cytosol, where it triggers the caspase cascade of apoptosis activation [47,48]. The release of Cyt c is regulated by members of the B-cell lymphoma 2 (Bcl-2) family, which includes both the pro-apoptotic Bcl-2-like protein 4 (Bax) and anti-apoptotic (Bcl-2) proteins [50]. As expected, our data show that the incubation of cardiomyocytes with Dox promotes a decrease in anti-apoptotic Bcl-2 protein expression and an increase in the pro-apoptotic Bax after a 48-h incubation. Conversely, in cells co-incubated with HT, no variation in the Bcl-2/Bax ratio was observed, thus suggesting that the presence of HT is able to interfere with the Bax pro-apoptotic pathway. This hypothesis has been further confirmed via analysis of caspase 3 activation in the same experimental groups. Indeed, while Dox promotes caspase 3 cleavage and p38 phosphorylation in a 48-h incubation, no activation has been observed in cells co-incubated with HT, thus suggesting that HT is able to prevent Dox-induced apoptosis in H9c2 cells. In addition, we found that HT counteracts Dox-induced DNA damage in cardiomyocytes, as indicated by a reduction in γ-H2AX protein levels. The same effect has been observed in several plant polyphenols, and it has been associated with their ability to interfere with oxidative stress, a key event underlying the interconnected intracellular pathways also involved in DNA damage [64,65,66,67,68,69] (Figure 9).

Recently, the protective effect of HT against Dox-induced cardiotoxicity was investigated in rats [39]. In this study, it was demonstrated that HT could effectively ameliorate Dox-induced heart damage by improving the mitochondrial electron transport chain and oxidative damage. Our results further support these in vivo data, as HT seems to counteract Dox-induced cardiotoxicity by strongly reducing Dox-mediated ROS production and oxidative stress. In addition, our data suggest that HT is able to inhibit the Bax pro-apoptotic pathway, thus interfering with caspase 3 activation and apoptosis.

The protective effect observed for HT in Dox-mediated oxidative stress could also be associated with its ability to chelate iron. Indeed, it has been recently observed that HT, being able to chelate iron, can attenuate the H_2_O_2_-induced and labile iron-mediated prolonged phosphorylation of JNK and p38, thus leading to mitochondrial destabilization, followed by cytochrome C release, caspase 3 activation and cell apoptosis [70]. In this respect, the lower concentration of iron could affect the ability of Dox to promote ROS production and apoptosis activation. Indeed, Dox possesses a strong affinity for iron, and the Dox-Fe^2+^ complex can promote lipid peroxidation by interacting with negatively charged membranes [5,71]. A reduction in Dox via free iron promotes the cycle of free radical formation (redox cycle), and recent evidence has shown that Dox’s effect on iron metabolism is mediated by proteins that sequester intracellular iron. Conversely, under physiological conditions, free iron is not sufficient to combine with Dox to cause cardiomyopathy, as the content of the free iron in most cells is very low [72]. Because impaired iron sequestration represents a critical component in Dox-induced cardiotoxicity, the ability of HT to sequester free iron could represent a further protection mechanism against the Dox-toxicity observed in cardiomyocytes. Moreover, our data also show that HT does not interfere with the antineoplastic properties of Dox in osteosarcoma cells, thus excluding possible impairment of Dox antitumor activity by HT.

## 5. Conclusions

The overall data suggest that HT is able to counteract Dox-induced cytotoxicity in cardiomyocytes by limiting both the oxidative response and apoptotic mechanisms. Although further investigation is needed to fully clarify the related molecular mechanisms, nevertheless, this study suggests that HT is able to protect cardiomyocytes against Dox-induced toxicity without affecting its antitumor activity. HT is characterized by a wide safety profile and is able to cross the blood–brain barrier. In this respect, our findings identify new beneficial properties for HT and suggest it might be a promising molecule for developing supplementary therapeutic approaches aimed at preventing anthracycline-related cardiotoxicity, as well as improving long-term outcomes in antineoplastic treatments.

## Data Availability

Data are contained within the article.
